# Designing advanced S‐scheme CdS QDs/La‐Bi_2_WO_6_ photocatalysts for efficient degradation of RhB

**DOI:** 10.1002/EXP.20230050

**Published:** 2023-08-21

**Authors:** Jing Ning, Bohang Zhang, Letu Siqin, Gaihui Liu, Qiao Wu, Suqin Xue, Tingting Shao, Fuchun Zhang, Weibin Zhang, Xinghui Liu

**Affiliations:** ^1^ School of Physics and Electronic Information Yan'an University Yan'an People's Republic of China; ^2^ Key Laboratory of Semiconductor Photovoltaic at Universities of Inner Mongolia Autonomous Region, School of Physical Science and Technology Inner Mongolia University Huhhot Inner Mongolia People's Republic of China; ^3^ Network Information Center Yan'an University Yan'an People's Republic of China; ^4^ Yunnan Key Laboratory of Opto‐Electronic Information Technology College of Physics and Electronics Information Yunnan Normal University Kunming People's Republic of China; ^5^ Department of Materials Science and Engineering City University of Hong Kong Kowloon Hong Kong People's Republic of China; ^6^ Department of Materials Physics Saveetha School of Engineering Saveetha Institute of Medical and Technical Sciences (SIMTS) Chennai Tamil Nadu India

**Keywords:** CdS quantum dots, density functional theory, La‐Bi_2_WO_6_, photocatalytic degradation, S‐scheme heterojunction

## Abstract

Finding effective strategies to design efficient photocatalysts and decompose refractory organic compounds in wastewater is a challenging problem. Herein, by coupling element doping and constructing heterostructures, S‐scheme CdS QDs/La‐Bi_2_WO_6_ (CS/LBWO) photocatalysts are designed and synthesized by a simple hydrothermal method. As a result, the RhB degradation efficiency of the optimized 5% CS/LBWO reached 99% within 70 min of illumination with excellent stability and recyclability. CS/LBWO shows improvement in the adsorption range of visible light and promotes electron–hole pair generation/migration/separation, attributing the superior degradation performance. The degradation RhB mechanism is proposed by a free radical capture experiment, electron paramagnetic resonance, and high‐performance liquid chromatography‐mass spectrometry results, indicating that h^+^ and •O_2_^–^ play a significant role during four degradation processes: de‐ethylation, chromophore cleavage, ring opening, and mineralization. Based on in situ irradiated X‐ray photoelectron spectroscopy, Mulliken electronegativity theory, and the work function results, the S‐scheme heterojunction of CS/LBWO promotes the transfer of photogenerated electron–hole pairs and promotes the generation of reactive radicals. This work not only reports that 5% CS/LBWO is a promising photocatalyst for degradation experiments but also provides an approach to design advanced photocatalysts by coupling element doping and constructing heterostructures.

## Graphical abstract

S‐scheme CdS QDs/La‐Bi_2_WO_6_ photocatalysts are successfully designed and synthesized by coupling element doping and constructing. The photocatalytic mechanism of the S‐scheme heterojunction was analyzed by the test of photocatalytic performance, crystal characterization and theoretical calculation, which provides an approach to design advanced photocatalysts by coupling element doping and constructing heterostructures.

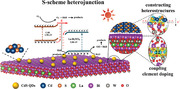

## 1 INTRODUCTION

With rapid population growth and extensive industrialization, the widespread discharge of various hazardous wastes and organic pollutants has made water pollution an increasingly severe problem.^[^ ^1^, ^2^, ^3^ ^]^ Among these pollutants, the dye concentration in water is more significant and has received greater attention. The standard sewage treatment methods include adsorption,^[^ ^4^, ^5^, ^6^ ^]^ coagulation,^[^ ^7^, ^8^ ^]^ oxidation,^[^ ^9^, ^10^ ^]^ electrolysis,^[^ ^11^, ^12^, ^13^ ^]^ and biological treatment methods.^[^ ^14^, ^15^ ^]^ However, these methods have several disadvantages, including high costs, long treatment cycles, and poor treatment efficiency.

Photocatalysis technology has the advantages of low cost, a friendly environment, and a wide application range and is considered an excellent method to solve environmental problems.^[^ ^16^ ^]^ Many scholars have conducted corresponding studies on TiO_2_,^[^ ^17^, ^18^, ^19^ ^]^ ZnO,^[^ ^20^, ^21^, ^22^, ^23^ ^]^ BiOX (X = I, Cl, Br),^[^ ^24^, ^25^, ^26^ ^]^ and Bi_2_WO_6_,^[^ ^27^, ^28^ ^]^ which have been widely used in the field of global environment governance. Notably, Bi_2_WO_6_, a simple class of N type semiconductor materials in the Aurivillius family, with a well‐visible response and suitable band gap (≈2.7 eV). Bi_2_WO_6_ is alternately composed of a (Bi_2_O_2_)^2+^ layer and a (WO_4_)^2‒^ layer and belongs to the rhombic crystal system. Although a single Bi_2_WO_6_ exhibits an excellent visible light response, the problems of low carrier separation efficiency and high recombination efficiency significantly limit its photocatalytic degradation of pollutants.^[^ ^29^ ^]^

Element‐doping^[^ ^30^ ^]^ and constructing heterostructures^[^ ^31^, ^32^ ^]^ are general strategies to enhance the photocatalytic performance of a single catalyst. For instance, Lee et al.^[^ ^33^ ^]^ studied Eu^3+^ doping on the photodegradation efficiency of Bi_2_WO_6_ samples. As a result, the incorporation of Eu^3+^ into Bi_2_WO_6_ and Eu^3+^ as an electron acceptor accelerated the transfer of photoinduced electrons in the Bi_2_WO_6_ crystal; thus, it improved the separation efficiency of photoinduced electron holes and enhanced the pollutant degrading capacity of *x*Eu‐Bi_2_WO_6_. Li et al.^[^ ^34^ ^]^ successfully synthesized Yb‐doped Bi_2_WO_6_ nanomaterials by a hydrothermal method. The results showed that the Bi_2_WO_6_ samples doped with Yb produced more active functional groups that promoted photocatalytic reactions, which hindered photogenerated electron‐hole pair recombination and improved the photodegradation performance. Our previous study modified Bi_2_WO_6_ material by doping La^3+^ ions.^[^ ^35^ ^]^ Compared with pure Bi_2_WO_6_, the particle size of La‐Bi_2_WO_6_ was reduced, and 2% La^3+^ doping effectively alleviates the phenomenon of easy recombination.

The interfacial effect between materials plays a powerful role in the improvement of photocatalytic reaction efficiency.^[^ ^36^ ^]^ Thus, constructing heterojunctions can effectively accelerate the separation and transfer of photocarriers.^[^ ^31^, ^37^ ^]^ For instance, semiconductor quantum dots ( QDs) due to their unique zero‐dimensional structure, many scholars have applied their to construct heterojunctions, such as CdS,^[^ ^38^ ^]^ CdSe,^[^ ^39^ ^]^ and PbSe.^[^ ^40^ ^]^ These QDs combined with TiO_2_ as heterostructures effectively improve the photocurrent response of intrinsic TiO_2_ and show good photocatalytic ability. Among these QDs, as a narrow band gap material, CdS has a good response range to visible light (*λ* < 520 nm), and its conduction band edge position is relatively negative, which is a kind of material with a high solar energy utilization rate.^[^ ^41^, ^42^ ^]^ For example, Ge et al.^[^ ^43^ ^]^ used a hydrothermal method to synthesize CdS QDs‐Bi_2_WO_6_, demonstrating the promising degradation efficiency of methyl orange due to the sensitization with CdS‐QD_S_ with the improvement of the migration efficiency of photogenerated charge carriers. Sun et al.^[^ ^44^ ^]^ successfully prepared the surface oxygen vacancy defect ZnO_1__‐x_‐TiO_2__‐x_ modified by CdS QDs in various methods. The results showed that a Z‐scheme heterojunction was formed between the two substances through modifying CdS QDs and the spatial separation efficiency of photogenerated electron holes was increased by creating synergies between oxygen vacancies and heterojunctions, which showed good photothermal and photocatalytic properties. An et al.^[^ ^45^ ^]^ successfully prepared a 3DOH ZnTiO_3_‐ZnO‐TiO_2_ multicomponent composite material using CdS QDs‐assisted modification. The heterostructure formed greatly improved the hydrogen production efficiency (≈300 times vs TiO_2_). Through the characterization and analysis of materials, the introduction of CdS QDs improves composites' visible light response ability and provides more transmission paths. At the same time, the study found that CdS QDs can produce interaction with composites, which extends the life of photogenerated carriers, promotes separation, and inhibits recombination. In summary, combining element doping and constructing heterostructures to build La‐Bi_2_WO_6_ and CdS QDs will be a promising approach to achieve excellent photocatalytic degradation performance.

Herein, CdS QDs/La‐Bi_2_WO_6_ (CS/LBWO) composite photocatalytic materials were prepared successfully. The photocatalytic materials were characterized and analyzed by experimental and theoretical approaches. Under the irradiation of a 500 W xenon lamp, using RhB pollution embodies its photocatalytic performance by measuring the degradation curve. The possible degradation mechanism of the photocatalyst was analyzed and discussed by materials characterization, high‐performance liquid chromatography‐mass spectrometry, and theoretical calculations.

## 2 EXPERIMENT

### 2.1 Catalyst preparation

CS/LBWO composite photocatalytic materials were prepared by a hydrothermal method. The chemical reagents used for the preparation were not subjected to purification treatment and were all analytical‐grade compounds. First, the 2% La‐Bi_2_WO_6_ material was prepared according to a previous work scheme (in Supporting Information).^[^ ^35^ ^]^ The prepared 2% La‐Bi_2_WO_6_ (0.698 g) nanosheets were uniformly dispersed in ethanol (80 mL), and then uniform suspension A was formed by ultrasonic treatment at room temperature using the ultrasonic instrument (*T*: 30 min, *P*: 100 W, *F*: 40 kHz). Then, an appropriate amount (determined by the mass ratio) of Cd (CH_3_COO)_2_•2H_2_O was added to solution A and magnetically stirred (60 min) to form solution B. Then, an equal quality of CH_4_N_2_S was added to solution B and the two substances were thoroughly mixed and stirred to obtain solution C. Finally, the turbid solution was transferred into polytetrafluoroethylene lining (100 mL). The hydrothermal reaction was kept at 120°C for 10 h, and the samples were washed several times and dried. The prepared sample was labeled *x*% CS/LBWO (*x* = 3, 5, 7, 9). In the preparation of intrinsic CdS QDs, 2% La‐Bi_2_WO_6_ material was not added in the above preparation process.

### 2.2 Characterization

To explain the relevant properties and characteristics of the prepared composites, we used a variety of characterization methods to test the prepared samples, to explain the reasons that promote the photocatalytic degradation efficiency. The characterization methods and detailed test parameters are described in the Supporting Information.

### 2.3 Photocatalytic experiments

To test the catalytic activity of the samples, we measured the photocatalytic activity by simulating the degradation of RhB under solar illumination. A xenon light source (*P*: 500 W) was used as the light source, and the band of light source was not processed. The photocatalyst (50 mg) and RhB solution (50 mL, *C*: 10 mg•L^‒1^) were mixed in the photocatalytic glass tube. The suspension was stirred without visible light irradiation (30 min) to achieve adsorption–desorption equilibrium. The light source was turned on, the refrigerating machine was used to control the reaction temperature (15°C), and the suspension (5 mL) was extracted at certain intervals. Finally, a UV‒vis spectrophotometer (UV1901PC) was used to test the supernatant (Wavelength range: 190–340 nm) and record the absorbance of pollutants.

### 2.4 Calculation method

VASP software^[^ ^46^ ^]^ and projected affix‐plane wave (PAW) pseudopotential^[^ ^47^ ^]^ were used for all calculations in this work. To better explain the influence of CdS QDs and La^3+^ doping on the electronic structure of Bi_2_WO_6_, the generalized gradient approximation of the Perdew–Burke–Ernzerhof scheme (GGA‐PBE) was used to describe the exchange‐correlation potential.^[^ ^48^ ^]^ At the same time, the Tkatchenko–Scheffler method was used to describe the van der Waals interaction between interfaces.^[^ ^49^ ^]^ For structural optimization, 520 eV was set as the truncation energy, and the related parameter setting of EDIFF and EDIFFG was 1 ×10^‒5^ eV and 0.01 eV•Å^‒1^, respectively.^[^ ^50^ ^]^ A 4 × 4 × 1 K‐point grid was used for structural optimization and other performance calculations. Figure S1 shows the constructed heterojunction model diagram.

## 3 RESULTS AND DISCUSSION

### 3.1 Characterization of photocatalysts

To confirm the synthesis accuracy of the prepared materials, an X‐ray diffraction (XRD) pattern was obtained (Figure S2A). As a result, prominent peaks of CdS QDs samples are observed at 2*θ* diffraction angles of 26.6°, 44.1°, and 52.1°, which match with the CdS crystal (111), (220), and (311) planes (JCPDS No. 80‐0019), respectively. Among them, the strongest peak is (111), and according to the Scherrer equation,^[^ ^51^ ^]^ the size of CdS prepared is approximately 15 nm, indicating the successful preparation of quantum dots. Moreover, the prominent peaks of the 2% La‐Bi_2_WO_6_ and CS/LBWO samples are observed at 2*θ* = 28.3°, 32.9°, 47.1° and 56°, which were matched with the (113), (200), (220), and (313) crystal planes (JCPDS No. 39‐0256), and the most substantial peaks were the (113) crystal planes. No other impurity peaks were detected, indicating the prepared sample had high crystallinity and purity. Notably, the lattice structure of 2% La‐Bi_2_WO_6_ did not change due to the introduction of quantum dots. At the same time, the diffraction peak of CdS QDs cannot be observed in the CS/LBWO composite. The small size and low compound content of CdS QDs mainly cause these phenomena.^[^ ^52^, ^53^ ^]^

**FIGURE 1:**
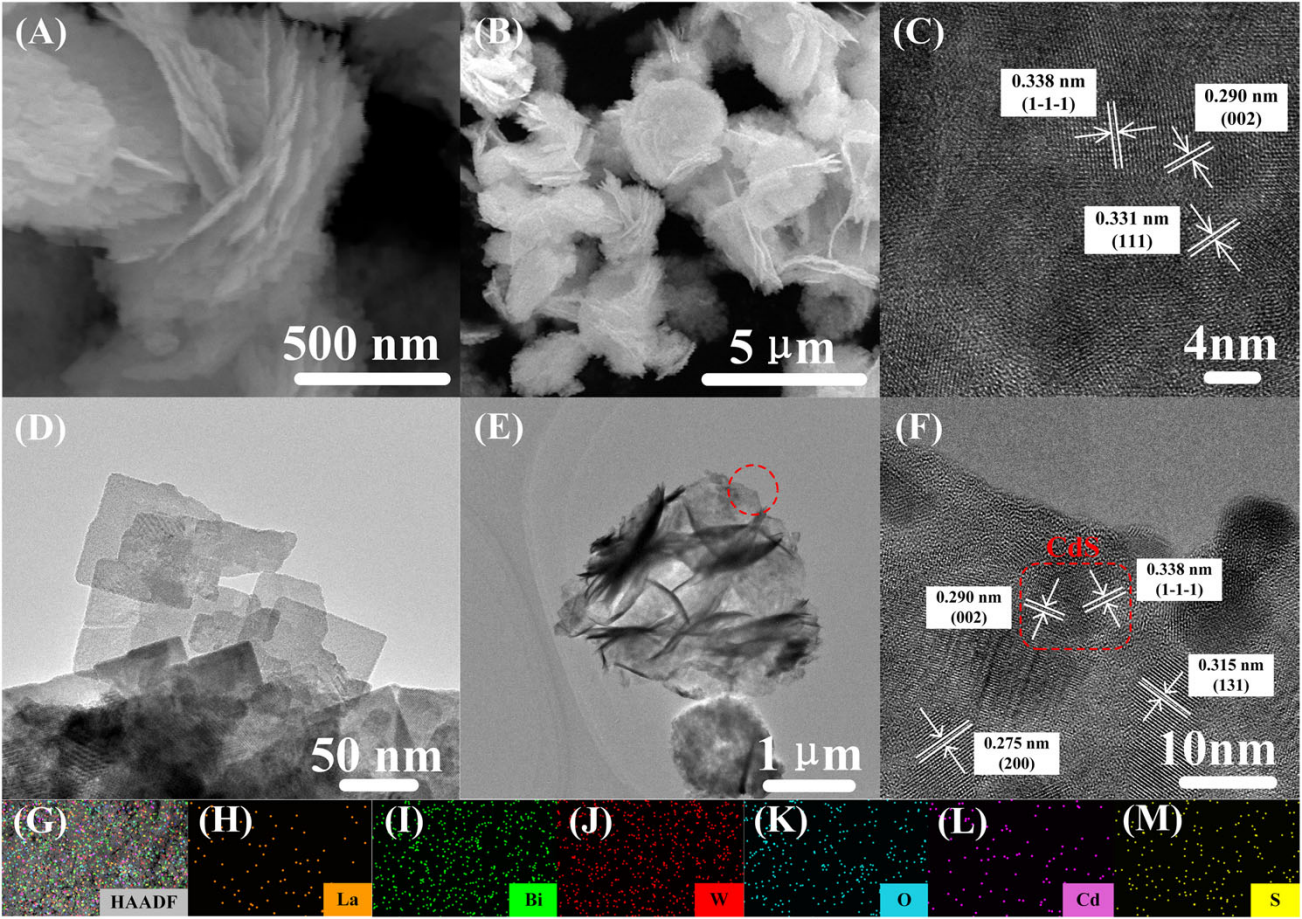
The microstructure information of catalyst. A SEM diagram and B TEM diagram of 2% La‐Bi_2_WO_6_; C HRTEM diagram of CdS QDs; D SEM diagram, E TEM diagram, and F HRTEM diagram of 5% CS/LBWO; G–M Mapping of 5% CS/LBWO.

Scanning electron microscopy (SEM) and transmission electron microscopy (TEM) were performed to analyze the microstructure information of the sample. As a result, the 2% La‐Bi_2_WO_6_ material (Figure 1A,B) shows a flower‐like shape formed by accumulating many nanosheets. Figure S2B and Figure 1C show the TEM and HRTEM image of CdS QDs, the prepared CdS QDs show a size of about 15 nm, and the (111) crystal planes (0.331 nm) and (002) crystal planes (0.290 nm) of CdS are found. It further indicates the successful preparation of CdS QDs, which is consistent with the XRD analysis. SEM and TEM images of 5% CS/LBWO (Figure 1D,E) show a layered structure formed by the stacking of nanosheets, which is similar to La‐Bi_2_WO_6_, indicating that the combination with CdS QDs will not affect the morphology of La‐Bi_2_WO_6_. However, CdS QDs are hardly observed, which may be because CdS QDs are too small to be observed, consistent with XRD. The HRTEM image of 5% CS/LBWO (Figure 1F) shows that the lattice fringes obtained correspond to 3.150 and 2.750 Å for the (131) and (200) planes of Bi_2_WO_6_, respectively. The (1‐1‐1) crystal plane of CdS QDs with a lattice fringe of 0.331 nm was also observed. Moreover, the lattice stripes of the two substances cross each other, proving that CdS QDs and 2% La‐Bi_2_WO_6_ heterojunctions were successfully formed. In addition, the EDS elemental mapping of the 5% CS/LBWO composite was examined (Figure 1G–M), showing that La, Bi, O, W, Cd, and S are uniformly distributed.

**FIGURE 2:**
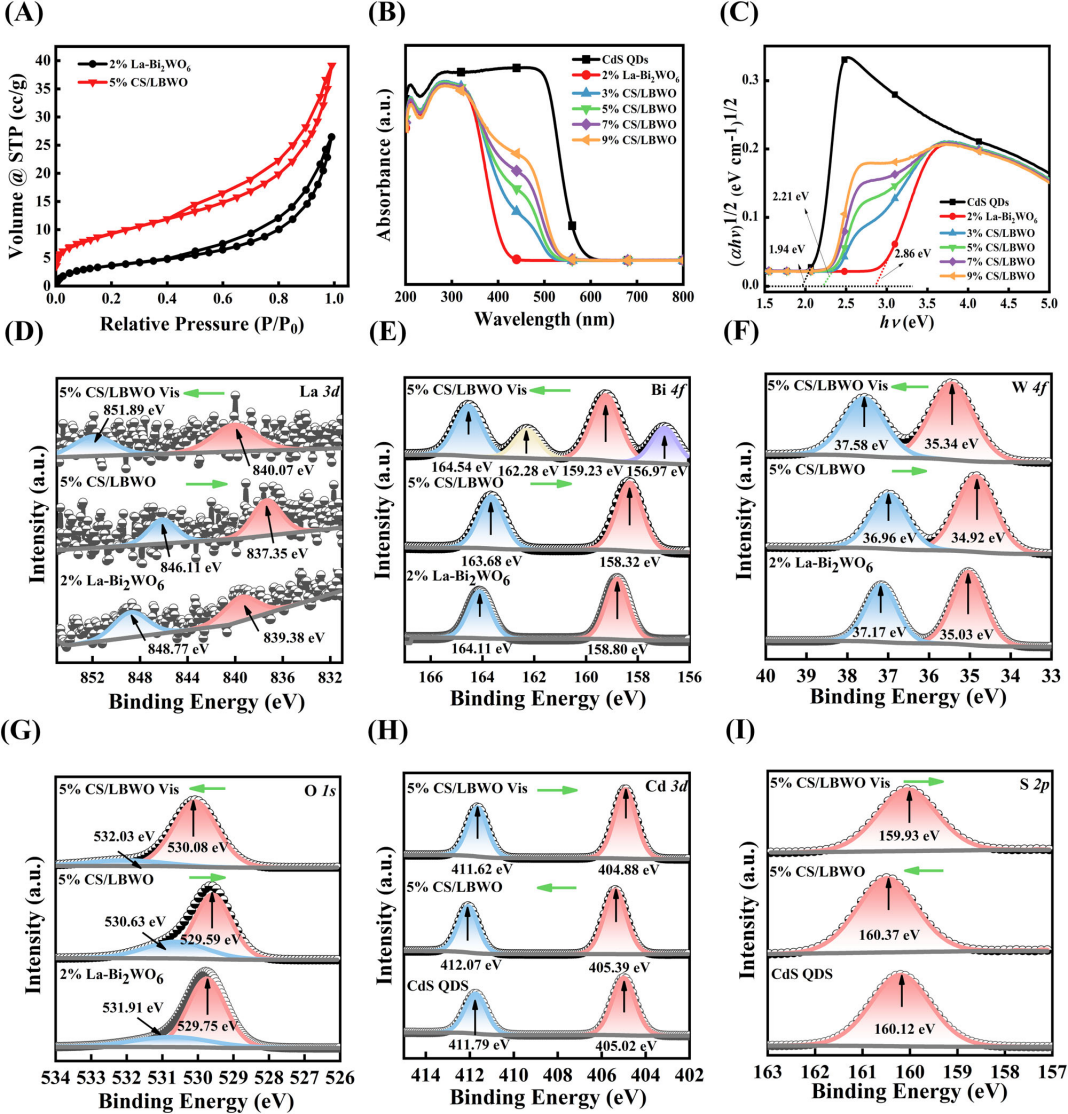
Surface, optical and elemental information of catalysts. A N_2_ physisorption and desorption isotherms of 2% La‐Bi_2_WO_6_ and 5% CS/LBWO samples; B UV–vis DRS absorption spectrum of CS/LBWO; C (αhμ)^1/2^ and hμ curves of CS/LBWO; The ISIXPS spectrum of 5% CS/LBWO D La 3d, E Bi 4f, F W 4f, G O 1s, H Cd 3d, I S 2p.

The specific surface areas for 2% La‐Bi_2_WO_6_ and 5% CS/LBWO were measured by N_2_ adsorption/desorption isotherms, and the pore size distributions were measured by the Barret–Joyner–Halenda method (BJH) (Figure 2A and Figure S3). The adsorption and desorption of the two materials correspond to type IV curves and H_3_ hysteresis loops.^[^ ^54^ ^]^ According to the pore size distribution diagram (Figure S3), 2% La‐Bi_2_WO_6_ shows a mesoporous structure, and the primary pore size is distributed between 3 and 20 nm. When *P*/*P*_0_ = 0.99034, the total pore volume is 0.041 cc•g^‒1^. After forming a heterojunction, the content of the pore size less than 10 nm increased significantly, the pore size of 2 nm was evenly distributed, and the total pore volume was 0.061 cc•g^‒1^ when *P*/*P*_0_ = 0.99142. The specific surface area, average pore size, and total pore volume for 2% La‐Bi_2_WO_6_ and 5% CS/LBWO are summarized in Table 1. As a result, the formation of heterojunctions between 2% La‐Bi_2_WO_6_ and CdS QDs can effectively increase the specific surface area, increase the pore volume, and decrease the pore diameter. A material with a large surface area is beneficial for increasing the number of adsorbed reaction sites of RhB, thereby improving the photocatalytic degradation efficiency.^[^ ^55^ ^]^

**TABLE 1:** Surface area, pore size, and pore volume of 2% La‐Bi_2_WO_6_ and 5% CS/LBWO.

Sample	Surface area (m^2^•g^‒1^)	Pore size (nm)	Pore volume (cc•g^‒1^)
2% La‐Bi_2_WO_6_	13.586	12.051	0.04092
5% CS/LBWO	33.442	7.258	0.06068

To determine the band gap and the light absorption characteristics for the current study material, UV–vis diffuse reflectance spectroscopy (UV‒vis DRS) tests were conducted, as shown in Figure 2B. The spectral response wavelengths of 2% La‐Bi_2_WO_6_ and CdS QDs are 430 and 580 nm, respectively. Compared with 2% La‐Bi_2_WO_6_, the wavelength of the spectral response of heterojunctions is shifted to the visible region, indicating its wider light absorption range. The corresponding band gap can be obtained by converting UV‒vis DRS into a Tauc diagram according to the Kubelka–Munk formula (Equation (1)):^[^ ^56^, ^57^ ^]^

(1) $$\alpha h\nu \;=\;A\;{\left(h\nu -E_{g}\right)}^{\frac{1}{2}}$$

α, *h*, and ν are the absorption/proportionality constant, Planck constant, and optical frequency, respectively. As a result, the band gap values of 3% CS/LBWO, 5% CS/LBWO, 7% CS/LBWO, and 9% CS/LBWO are 2.254 eV, 2.217 eV, 2.162 eV, and 2.135 eV, respectively (Figure 2C). The results show that suitable CdS QDs can strongly reduce the band gap of 2% La‐Bi_2_WO_6_, improve the corresponding range of visible light, promote electron and hole migration, and effectively enhance photocatalytic performance.

To analyze the element binding energy and the change of chemical environment on the catalyst, in situ irradiated X‐ray photoelectron spectroscopy (ISIXPS) was performed. Figure S4A shows that the 5% CS/LBWO sample contains La, Bi, W, O, Cd, and S, indicating that CdS QDs and La‐Bi_2_WO_6_ successfully form heterojunctions. This is consistent with the SEM results. C 1s standard peak (284.81 eV) was used for line calibration the survey spectra (Figure S4B), the peak may be caused by the contamination of carbon materials on the sample surface.^[^ ^58^, ^59^, ^60^ ^]^ In 5% CS/LBWO, the peaks of the La 3d_3/2_ and La 3d_5/2_ at 846.11 eV and 837.35 eV, confirming that La^3+^ had been successfully doped (Figure 2D).^[^ ^61^ ^]^ The Bi 4f of samples at 163.68 eV and 158.32 eV are attributed to two spin orbitals of Bi 4f_5/2_ and Bi 4f_7/2_ (Figure 2E), indicating Bi presents trivalent oxidation state in 5% CS/LBWO.^[^ ^62^ ^]^ The peaks at 36.96 eV and 34.92 eV for W 4f_5/2_ and W 4f_7/2_ are matched to W^6+^ (Figure 2F).^[^ ^63^, ^64^ ^]^ The characteristic peaks of O 1s in the 5% CS/LBWO sample are 530.63 eV and 529.59 eV, corresponding to the Bi─O bond and W─O bond in the (Bi_2_O_2_)^2+^ and (WO_4_)^2–^ lattices (Figure 2G).^[^ ^65^, ^66^ ^]^ The peaks of Cd 3d_3/2_ and Cd 3d_5/2_ were observed at 412.06 and 405.39 eV, respectively (Figure 2H), indicating the bivalence of Cd in 5% CS/LBWO.^[^ ^67^ ^]^ The S‐element binding energy of 5% CS/LBWO is 160.37 eV, which corresponds to the characteristic peak of S 2p (Figure 2I).^[^ ^30^, ^68^ ^]^ The shift of the XPS peak corresponds to the change in binding energy, which can be used to reflect and judge the direction of the electron. When a material gains electrons, its elemental binding energy will decrease. Conversely, if it loses electrons, its elemental binding energy will increase. Compared to 2% La‐Bi_2_WO_6_, the characteristic peaks of La 3d, Bi 4f, W 4f, and O 1s of 5% CS/LBWO are slightly shifted in the direction of lower energy. However, both Cd 3d and S 2p characteristic peaks for 5% CS/LBWO are shifted toward a high energy. Notably, each element binding energy of 5% CS/LBWO produced an opposite change after illumination. Among them, Bi 4f produces new characteristic peaks, which may be caused by the excitation of visible light and ionization. These results indicate that the electron transfer is generated between 2% La‐Bi_2_WO_6_ and CdS QDs, which promotes the separation of photogenerated electrons by the excitation of visible light.

**FIGURE 3:**
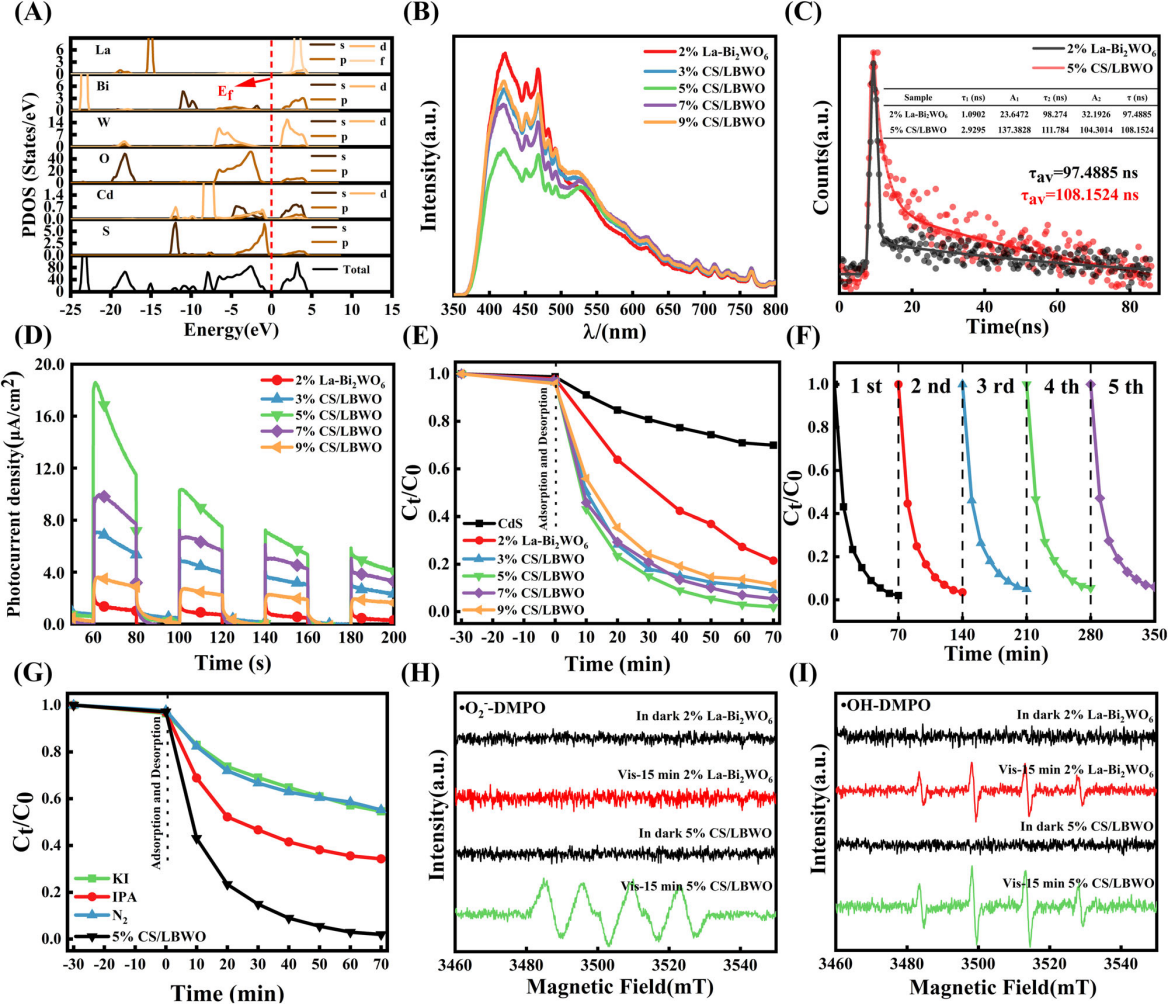
Electronic structure information and photocatalytic degradation experiment of catalysts. A The TDOS and PDOS of CS/LBWO; B The PL spectrum of 2% La‐Bi_2_WO_6_ and CS/LBWO; C Time‐resolved PL spectra of 2% La‐Bi_2_WO_6_ and CS/LBWO; D The photocurrent response of 2% La‐Bi_2_WO_6_ and CS/LBWO; E Photocatalytic degradation efficiency of CS/LBWO; F The cycle degradation efficiency diagram of 5% CS/LBWO; G Free radical capture diagram of 5% CS/LBWO; H EPR responses of 2% La‐Bi_2_WO_6_ and CS/LBWO to •O_2_^−^‐DMPO; I EPR responses of 2% La‐Bi_2_WO_6_ and CS/LBWO to •OH‐DMPO.

DFT calculations further studied the electronic structure of CS/LBWO heterojunction materials (Figure S5). The partial density of states (PDOS) and total density of states (TDOS) of CS/LBWO can show the variation of the band gap and the contribution of the element's electronic states (Figure 3A). According to TDOS, the theoretical band gap value of the CS/LBWO heterojunction is 1.954 eV. Meanwhile, compared with previous DFT studies on La‐Bi_2_WO_6_, the band gap decreases after forming a heterojunction with CdS QDs, consistent with UV‒vis DRS test results. As a result, the experimental and theoretical band gap values of both samples are summarized in Table 2. Specifically, the conduction band minimum (CBM) of the heterostructure mainly comes from the electronic state of La‐Bi_2_WO_6_, while the electronic state of CdS occupies the valence band maximum (VBM). In the CS/LBWO heterostructure, the valence band (VB) region (‒8 eV to 0 eV) is mainly contributed by O 2p, W 5d, Cd 4d, and S 3p. The conduction band (CB) region (0 eV to 5 eV) is mainly contributed by La 4f, Bi 6p, O 2p, and W 5d hybrid orbitals, and Cd 5s has a small contribution.

**TABLE 2:** Experimental and theoretical results of the band gap between the 2% La‐Bi_2_WO_6_ and 5% CS/LBWO samples.

Sample	Experimental values (eV)	Theoretical values (eV)
2% La‐Bi_2_WO_6_	2.864	2.792^[^ ^35^ ^]^
5% CS/LBWO	2.217	1.954

Photoluminescence spectroscopy (PL) is a common approach to observing recombination rates, since PL intensity is positively correlated with the separation efficiency of electron–hole pairs in materials.^[^ ^69^ ^]^ The characteristic peak range is between 400 and 550 nm, and significant firm peaks are detected at 420 and 450 nm (Figure 3B). Note that the PL strength of the CS/LBWO heterojunction is lower than that of 2% La‐Bi_2_WO_6_. Among them, 5% CS/LBWO has the lowest intensity, indicating effective inhibition of recombination of electron–hole pairs. Time‐resolved fluorescence decay spectra were also tested to further analyze the lifetime information of photogenerated charge carriers (Figure 3C). The double exponential decay model is used to fit the fluorescence attenuation data (Equation (2)), the lifetime and the coefficients could be obtained after fitting (*A*_1_,$\;A_{2}$,$\;\tau_{1}$, τ_2_). The short‐lifetime τ_1_ and long‐lifetime τ_2_ represent the radiative process of carrier recombination and the non‐radiative energy transfer process, respectively. Then the average lifetime (τ) of carriers before and after recombination by Equation (3).^[^ ^70^ ^]^

(2) $$y=y_{0}+A_{1}e^{-x/\tau_{1}}+A_{2}e^{-x/\tau_{2}}$$

(3) $$\tau =\frac{A_{1}\tau_{1}^{2}+A_{2}\tau_{2}^{2}}{A_{1}\tau_{1}+A_{2}\tau_{2}}$$

The τ_1_ and τ_2_ of 5% CS/LBWO (2.929 ns and 111.784 ns) are both improved compared with 2% La‐Bi_2_WO_6_ (1.090 ns and 98.274 ns) and the average life is also improved (97.4885 ns to 108.1524 ns). The above results show that the recombination of electron–hole pairs can be effectively inhibited and the lifetime of photogenerated charge carriers can be increased by constructing heterojunction with CdS QDs, which is consistent with the previous analysis.

The photocurrent response is positively correlated with the visible light utilization capacity of the material. As a result, the photocurrent response test (Figure 3D) shows a trend similar to that of the PL spectra. Compared with 2% La‐Bi_2_WO_6_, the photocurrent response of CS/LBWO is significantly enhanced. The photocurrent response of 5% CS/LBWO is the highest among the prepared catalysts, illustrating its promising application for photocatalytic degradation reactions. Meanwhile, the Nyquist conversion diagram of EIS can reflect the charge transfer resistance, indicating the degree of a compound of electron–hole pairs.^[^ ^71^ ^]^ The EIS conversion diagram (Figure S6) shows that the arc radius of the CS/LBWO electrode is smaller because the CdS QDs compound reduces the electron transfer resistance, promotes the charge transfer between the heterojunction, and inhibits electron–hole pair recombination. In summary, according to PL, photocurrent response, and EIS tests, we found that the construction of heterojunction CS/LBWO can promote the generation of electron–hole pairs, promote separation and inhibit recombination, thus leading to the improvement of its photocatalytic degradation efficiency.

### 3.2 Photocatalytic degradation experiment

The photocatalytic degradation ability of the current study samples was tested by RhB under simulated sunlight irradiation. The photodegradation rates of CdS, 2% La‐Bi_2_WO_6_, 3% CS/LBWO, 5% CS/LBWO, 7% CS/LBWO, and 9% CS/LBWO are 30%, 78%, 92%, 99%, 94%, and 89% in 70 min (Figure 3E), respectively. As expected, 5% CS/LBWO shows superior degradation performance compared to the others. At the same time, we compared with other similar heterojunction work, as detailed in Table S1. The S‐scheme CdS QDs/La‐Bi_2_WO_6_ photocatalyst performs well in many heterojunctions, which indicates a good development prospect. To further verify the degradation effect of the photocatalyst, the experiment of total organic carbon test provides information to analyze the mineralization of pollutants. The content of total organic carbon (TOC) is calculated by the difference between total carbon (TC) and total inorganic carbon (TIC) (TOC = TC − TIC). The results are shown in Table 3 and Table S2. The TOC removal rates of 2% La‐Bi_2_WO_6_ and 5% CS/LBWO systems are 73.01% and 87.92% in 70 min (note that the removal rate = (TOC (0 min) ‒ TOC (*x* min)) /TOC (0 min)). It further shows that the formation of heterojunction system by CdS QDs can effectively promote the degradation and mineralization of pollutants.

**TABLE 3:** TOC removal rate of 2% La‐Bi_2_WO_6_ and 5% CS/LBWO.

Sample (Time)	TIC (mg•L^‒1^)	TC (mg•L^‒1^)	TOC (mg•L^‒1^)	Removal rate
2% La‐Bi_2_WO_6_ (0)	0.637	18.731	18.094	∖
2% La‐Bi_2_WO_6_ (70)	3.765	8.647	4.882	73.01%
5% CS/LBWO (0)	0.608	18.560	17.952	∖
5% CS/LBWO (70)	3.254	5.421	2.167	87.92%

To study the recycling ability of the 5% CS/LBWO photocatalyst, this catalyst was recycled five times under the same reaction conditions (Figure 3F), indicating that the RhB degradation efficiency of 5% CS/LBWO is maintained at ≈94%, which is promising for practical application. Before and after the degradation, XRD and SEM were studied to gain insight into the superior stability (Figures S7 and S8) and the concentration of the Cd^2+^ ions was detected by Inductively coupled plasma‒mass spectrometry (ICP‒MS) (Table S3). The XRD and SEM results show that the changes in morphology and phase after photocatalytic degradation are not significant. And the ICP‒MS indicates that appropriate Cd^2+^ ions can promote the enhancement of photocatalytic efficiency and Cd^2+^ can effectively recover by the use of bentonite. These results indicate that 5% CS/LBWO photocatalyst has high photocatalytic activity and good recyclability in the degradation process.

Free radical capture experiments were studied to describe the active ingredients involved in the reaction of 5% CS/LBWO. Holes (h^+^), superoxide radicals (•O_2_^–^), and hydroxide radicals (•OH) are the dominant active free radical substances during the photocatalytic process. Thus, 1 mmol KI (capture h^+^), 1 mmol IPA (capture •OH), and N_2_ (capture •O_2_^–^) were added to the reaction system. As shown in Figure 3G, photocatalytic degradation was significantly inhibited after adding trapping agents, showing that the three active substances were involved in the degradation of RhB. Note that h^+^ and •O_2_^–^ play significant roles in comparison with •OH. To further investigate the effect of constructing heterojunction on the formation of free radicals, the catalyst was studied by the electron paramagnetic resonance (EPR) method under simulated sunlight irradiation. The •O_2_^–^ and •OH are detected by the DMPO trapping agent. Figure 3H,I show the responses of 2% La‐Bi_2_WO_6_ and 5% CS/LBWO to •O_2_^–^‐DMPO and •OH‐DMPO before and after illumination. For the dark conditions, all materials are not observed with the characteristic peak of •O_2_^–^ and •OH. After 15 min of light, 2% La‐Bi_2_WO_6_ observed the production of •OH free radicals, not •O_2_^–^ free radicals. Notably, the characteristic peaks of •O_2_^–^ and •OH can be observed in 5% CS/LBWO, and the characteristic peak of •OH free radicals is higher. These results show that more active free radicals can be produced to promote photocatalytic reactions by constructing a heterojunction with CdS QDs.

**FIGURE 4:**
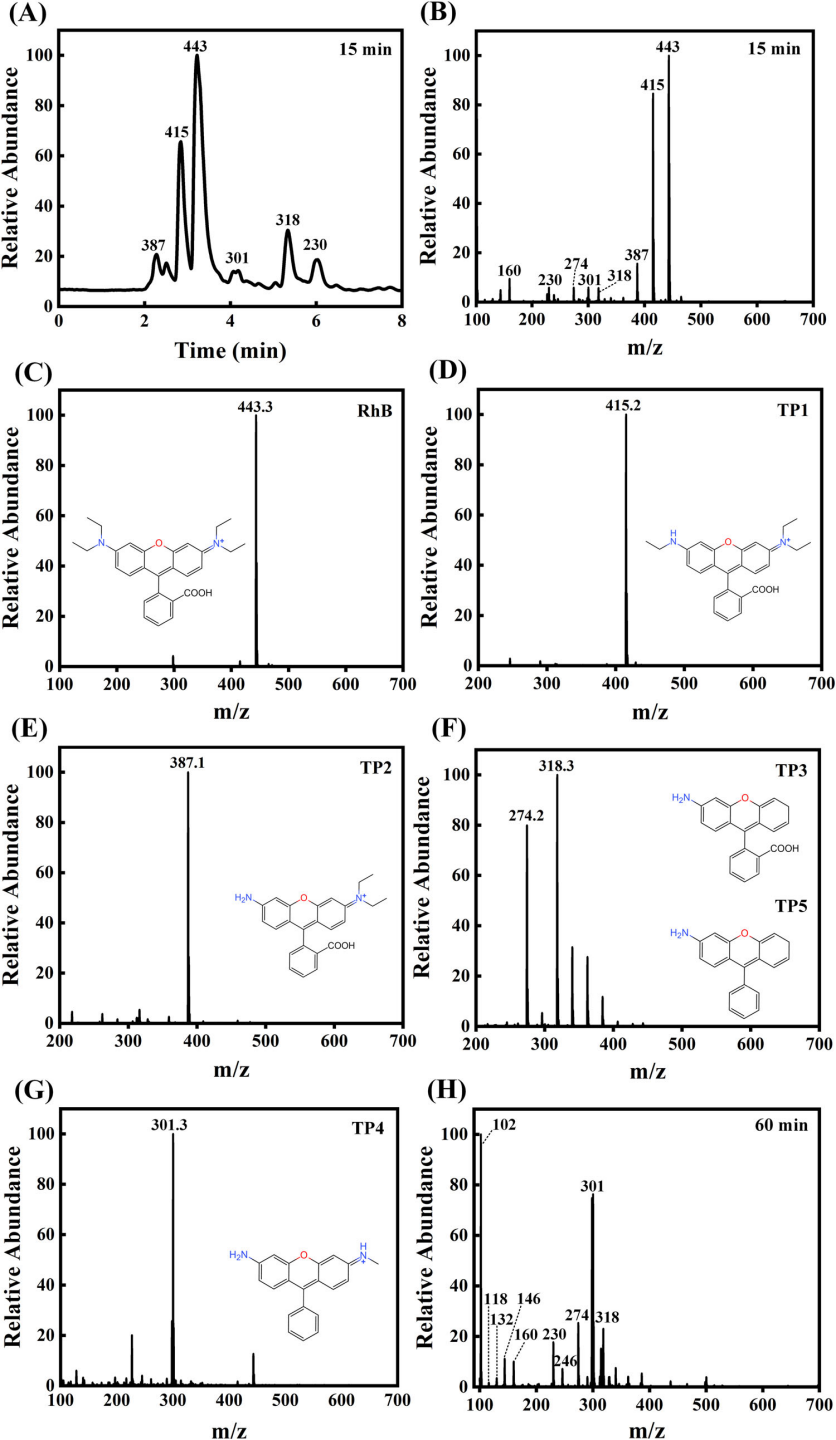
The HPLC‒MS experiment of CS/LBWO. A Chromatography of intermediates after 15 min; B Primary mass spectrometry of intermediates after 15 min; C–G secondary mass spectrometry of intermediates after 15 min; H Primary mass spectrometry of intermediates after 60 min.

To further analyze the mineralization process and photocatalytic degradation mechanism for 5% CS/LBWO, HPLC‒MS analyses were performed on the contaminant solutions after the reaction for 15 and 60 min. According to the chromatographic diagram (Figure 4A) and primary mass spectrometry (Figure 4B), RhB (*m/z* = 443, *t_R_* = 3.225 min) and five intermediates in the solution are easily found after 15 min reaction. According to the secondary mass spectrometry (Figure 4C–G), the structural formulas of the intermediate products are C_26_H_27_O_3_N_2_^+^ (TP1, *m/z* = 415, *t_R_* = 2.846 min), C_24_H_23_O_3_N_2_^+^ (TP2, *m/z* = 387, *t_R_* = 2.276 min), C_20_H_15_O_3_N (TP3, *m/z* = 318, *t_R_* = 5.339 min), C_20_H_17_ON_2_^+^ (TP4, *m/z* = 301, *t_R_* = 4.122 min), C_19_H_15_ON (TP5, *m/z* = 274, *t_R_* = 5.319 min), respectively. As the reaction continues, the intermediate products split further. According to the first‐order mass spectrometry (Figure 4H), the peak of RhB disappeared, and many new small molecule products were generated after 60 min reaction. The structural of the small molecule products are pimelic acid (*m/z* = 160), adipic acid (*m/z* = 146), glutaric acid (*m/z* = 132), succinic acid (*m/z* = 118), valeric acid (*m/z* = 102), respectively.

**FIGURE 5:**
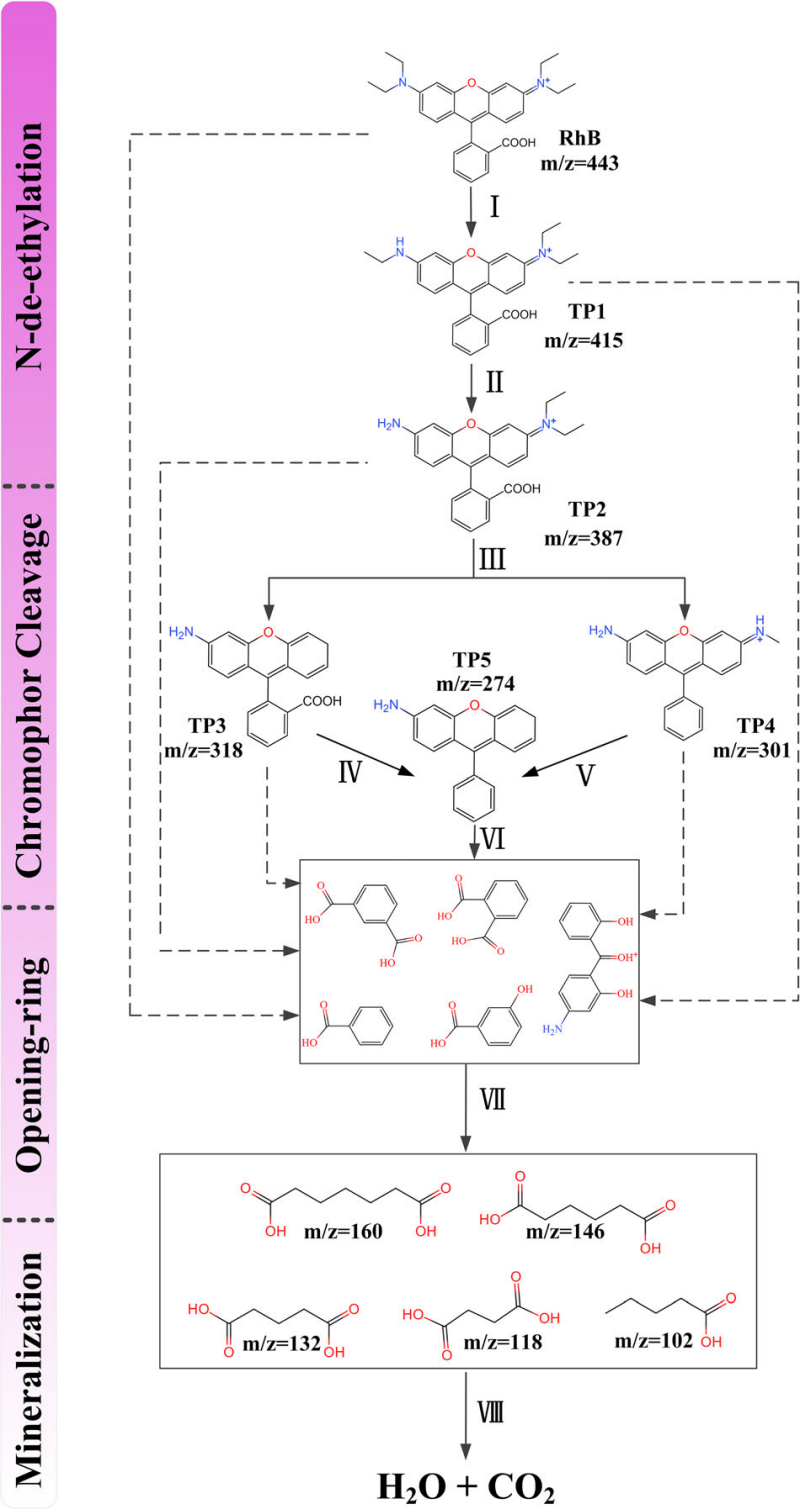
Possible photocatalytic degradation pathways of RhB.

Based on the results mentioned above, we propose a possible pathway for CS/LBWO to degrade RhB in visible light response. Specifically, RhB is first attacked by h^+^ to produce TP1 and TP2 by two consecutive de‐ethylation, corresponding to the RhB de‐ethylation process (steps I and II). Subsequently, there are two cleavage pathways of TP2. The first is to generate TP3 by removing ─NH(C_2_H_5_)_2_, and the second is to generate TP4 by removing ─C_2_H_5_ and ─COOH. Then, TP3 removes ─COOH, and TP4 removes ─CH_4_N^+^ to produce TP5. According to some previous studies,^[^ ^72^, ^73^ ^]^ the intermediates of phthalic acid, m‐phthalic acid, m‐hydroxybenzoic acid, benzoic acid, and C_13_H_12_O_3_N^+^ (*m/z* = 230)^[^ ^74^ ^]^ are produced in the above cracking processes, corresponding to the chromophore cleavage process of RhB (steps III, IV, V, and VI). Then, a series of small molecule acyclic compounds are formed by the opening‐ring reaction of the intermediate (step VII). Finally, the small molecule products are mineralized to produce H_2_O and CO_2_ (step VIII). Overall, according to the plain chromatographic diagram, it is found that de‐ethylation, chromophore cleavage, ring opening, and mineralization coexisted in the degradation process (Figure 5).

### 3.3 Investigation of the mechanism of photocatalytic degradation

The energy band structure and photocatalytic activity enhancement mechanism were studied to explore the synergistic interaction between the heterojunction of CS/LBWO. The formed types of CS/LBWO heterojunctions can be judged by the position of the VB and CB, which can be determined by the following equation (Equations (4), (5), and (6); Mulliken electronegativity theory):^[^ ^75^ ^]^

(4) $$E_{VB}=\chi -E_{e}+0.5E_{g}$$

(5) $$E_{CB}=E_{VB}-E_{g}$$

(6) $$\chi \;=\;{\left[\chi {\left(A\right)}^{a}\chi {\left(B\right)}^{b}\chi {\left(C\right)}^{c}\right]}^{1/\left(a+b+c\right)}$$

*E_VB_* is the energy position of the VB, $E_{g}$ is the bandgap obtained from the experiment, *E_CB_* is the energy position of the CB, *E_e_* is the energy of a free electron at a hydrogen scalar potential (*E_e_* = 4.5 eV). Moreover, *χ* is the absolute value of electronegativity, *χ*(*A*), *χ*(*B*), and *χ*(*C*) is the absolute value of the electronegativity of a single element semiconductor and *a*, *b*, and *c* are the coefficients of the corresponding elements in the structural formula. According to the calculation, the positions of the VB for CdS QDs and La‐Bi_2_WO_6_ are +1.225 eV and +3.134 eV, respectively. The CB positions of CdS QDs and La‐Bi_2_WO_6_ are −0.715 eV and +0.274 eV, respectively. We find that the CB and VB of CdS QDs are lower than those of La‐Bi_2_WO_6_. According to the energy band structure of semiconductors, many styles of possible formed heterojunctions between La‐Bi_2_WO_6_ and CdS QDs.^[^ ^76^ ^]^ The main difference between the types of heterojunctions is the direction of electron migration.

The electron transfer direction of CS/LBWO is analyzed to further determine the heterojunction type. The work functions (*Φ*) of CdS QDs (111) and La‐Bi_2_WO_6_ (110) were calculated according to the following Equation (7):^[^ ^77^ ^]^

(7) $$\Phi \;=\;E_{\mathrm{VAC}}-E_{f}$$

**FIGURE 6:**
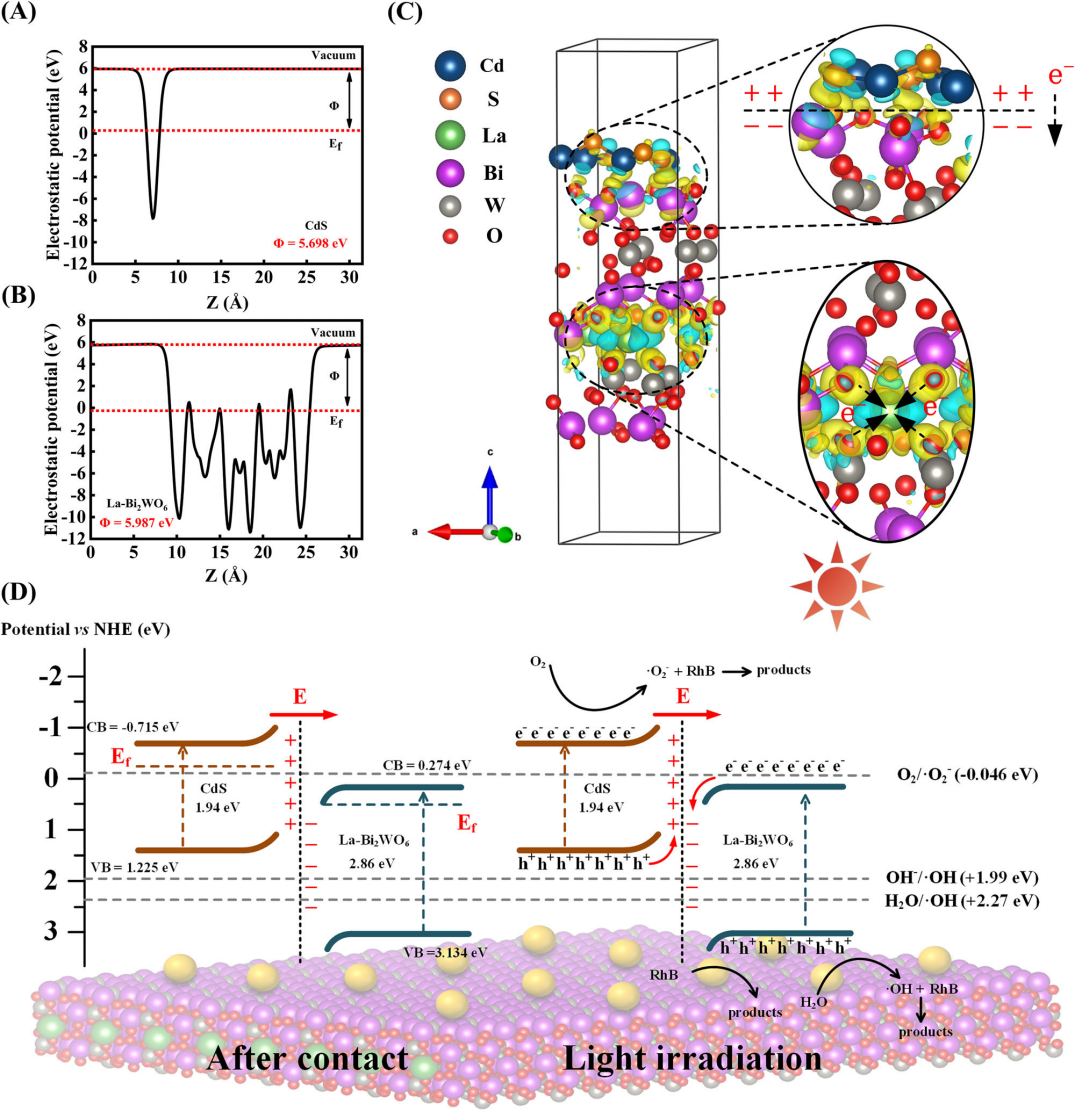
The scheme determination and photocatalytic mechanism analysis of CS/LBWO heterojunction. A Surface work functions of CdS; B Surface work functions of La‐Bi_2_WO_6_; C Three‐dimensional differential charge density diagram for CS/LBWO; Note that yellow parts represent electron loss and blue parts represent electron gain; D The photocatalytic mechanism of CS/LBWO S‐scheme heterojunction.

*E_vac_* is the electrostatic potential at the vacuum level, and *E_f_* is the Fermi level. *Φ* (CdS QDs (111)) and *Φ* (La‐Bi_2_WO_6_ (110)) are 5.698 eV and 5.987 eV (Figure 6A,B), indicating that the Fermi energy level of CdS QDs is higher than that of La‐Bi_2_WO_6_. Therefore, after forming a heterojunction between CdS QDs and La‐Bi_2_WO_6_, electrons tend to transfer from CdS QDs with a high Fermi level to the La‐Bi_2_WO_6_ surface with a low Fermi level to balance the Fermi energy,^[^ ^78^ ^]^ in good agreement with the conclusion of ISIXPS. The three‐dimensional differential charge density diagram of CS/LBWO (Figure 6C) shows that blue is concentrated near the La atoms and yellow surrounds the O atom, indicating that electrons are mainly transferred from Bi_2_WO_6_ to La. And due to the contact between CdS QDs and La‐Bi_2_WO_6_, electrons spontaneously transfer from CdS QDs to La‐Bi_2_WO_6_, resulting in an electric potential difference between them.^[^ ^1^ ^]^ It is consistent with the work function and ISIXPS analysis.

According to the materials characterization and DFT calculation results, CS/LBWO with S‐scheme heterojunction is proposed (Figure 6D). When surfaces of CdS QDs and La‐Bi_2_WO_6_ come into contact, since the Fermi energy level on the surface of CdS QDs is higher than that on the surface of La‐Bi_2_WO_6_, electrons transition from CdS QDs to La‐Bi_2_WO_6_. The surface of CdS QDs forms an electron depletion layer (positively charged) and the surface of La‐Bi_2_WO_6_ forms an electron accumulation layer (negatively charged); then, an internal electric field is created from CdS QDs to La‐Bi_2_WO_6_.^[^ ^79^, ^80^ ^]^ Thus, the binding energies of Cd and S are decreased and La, Bi, W, and O are increased.

Under the irradiation of visible light, La‐Bi_2_WO_6_ and CdS QDs produce positively charged holes and negatively charged highly active electrons in the VB and CB, respectively. Because of the internal electric field, the photogenerated electrons in CB of La‐Bi_2_WO_6_ are transferred to VB of CdS QDs. This phenomenon leads to an opposite change in the binding energy of the elements before and after illumination. Then the standard redox potential of O_2_/•O_2_^–^ (–0.046 eV) is lower than the CB of La‐Bi_2_WO_6_, e^–^ cannot reduce O_2_ to •O_2_^–^. Similarly, the standard redox potential of H_2_O/•OH (+2.27 eV) is higher than the VB of CdS QDs, so the h^+^ of CdS QDs cannot oxidize H_2_O to •OH. The oxidation potential of La‐Bi_2_WO_6_ (+3.134 eV) is higher than that of H_2_O/•OH (+2.27 eV) and OH^–^/•OH (+1.99 eV), so the holes in the VB of La‐Bi_2_WO_6_ are sufficient to oxidize H_2_O and OH^–^ to generate •OH and can also directly participate in the degradation process of RhB. Subsequently, RhB undergoes four stages for de‐ethylation, chromophore cleavage, ring opening, and mineralization under the coaction of h^+^, •O_2_^–^, and •OH. Finally, degradation into H_2_O and CO_2_. The synergistic effect between the S‐scheme heterojunction and La doped can effectively improve the separation and recombination of photogenerated electron–hole pairs and greatly enhance the photocatalytic degradation ability of Bi_2_WO_6_.^[^ ^81^ ^]^

## 4 CONCLUSIONS

In summary, CS/LBWO S‐scheme heterojunction was successfully constructed using element doping and quantum dot modification and showed excellent photocatalytic activity under visible light. The heterojunction created by CdS QDs and La‐Bi_2_WO_6_ increases the specific surface area, decreases the pore size, and further enhances the photocatalytic reaction site, as evidenced by SEM, TEM, BET, and experimental degradation results. Furthermore, UV‒vis DRS analysis, PL, time‐resolved fluorescence, photocurrent response, and DOS simulation show improvement in visible light's adsorption range, electron–hole pair generation/migration/separation promotion, and photocatalytic performance for the formation of the heterostructure. Notably, the RhB degradation efficiency of 5% CS/LBWO reaches 99% after 70 min of illumination with excellent mineralization ability, stability, and recyclability, which is superior to others. Meanwhile, a possible RhB degradation mechanism is proposed by a free radical capture experiment and HPLC‒MS results, indicating that CS/LBWO mainly produces h^+^ and •O_2_^–^ functional groups to participate in photocatalytic degradation under visible light irradiation, proving the degradation of RhB through four processes: de‐ethylation, chromophore cleavage, ring opening, and mineralization. In addition, based on in situ irradiated X‐ray photoelectron spectroscopy, Mulliken electronegativity theory, and the work function results, the S‐scheme heterojunction of CS/LBWO is verified and described, which promotes the transfer of photogenerated electron–hole pairs and promotes the generation of reactive radicals. This work not only reports that 5% CS/LBWO is a promising photocatalyst for degradation experiments but also provides an approach by coupling element doping and constructing heterostructures to design advanced photocatalysts.

## CONFLICT OF INTEREST STATEMENT

The authors declare no conflicts of interest.

## Supporting information

Supporting information

## Data Availability

All data of this work are present in the article and the Supporting Information. The other data that support the findings of this work are available from the corresponding author upon reasonable request.
