# Supplementary material for: Designing advanced S‐scheme CdS QDs/La‐Bi2WO6 photocatalysts for efficient degradation of RhB
Source: Exploration (Beijing). 2023 Aug 21;3(5):20230050. doi: 10.1002/EXP.20230050 (PMC10582608; doi:10.1002/EXP.20230050)
Supplement: Supplementary file 1 — Supporting information [file EXP2-3-20230050-s001.docx]

**Supplementary material**

**Designing advanced S-scheme CdS QDs/La-Bi_2_WO_6_ photocatalysts for efficient degradation of RhB**

Jing Ning^1†^ **|** Bohang Zhang^1†^ **|** Letu Siqin^2^ **|** Gaihui Liu^1^ **|** Qiao Wu^1^ **|** Suqin Xue^1^ **|** Tingting Shao^1^ **|** Fuchun Zhang^1^ **|** Weibin Zhang^3^ **|** Xinghui Liu^4^

^1^ School of Physics and Electronic Information, Yan'an University, Yan'an 716000, China

^2^ Key Laboratory of Semiconductor Photovoltaic at Universities of Inner Mongolia Autonomous Region, School of Physical Science and Technology, Inner Mongolia University, 235 West University Road, Huhhot, Inner Mongolia 010021, China

^3^ College of Physics and Electronics Information, Yunnan Key Laboratory of Opto-Electronic Information Technology, Yunnan Normal University, Kunming, 650500, China

^4^ Department of Materials Science and Engineering, City University of Hong Kong, 83 Tat Chee Avenue, Kowloon 999077, Hong Kong, China; Department of Materials Physics, Saveetha School of Engineering, Saveetha Institute of Medical and Technical Sciences (SIMTS), Thandalam, Chennai 602105, Tamilnadu, India.

†Jing Ning and Bohang Zhang contributed equally.

**Correspondence**

1: Fuchun Zhang, School of Physics and Electronic Information, Yan'an University, Yan'an 716000, China.

E-mail: [yadxzfc@yau.edu.cn](mailto:yadxzfc@yau.edu.cn)

2: Weibin Zhang, College of Physics and Electronics Information, Yunnan Key Laboratory of Opto-Electronic Information Technology, Yunnan Normal University, Kunming 650500, China.

E-mail: [220001@ynnu.edu.cn](mailto:220001@ynnu.edu.cn)

3: Xinghui Liu, Department of Materials Science and Engineering, City University of Hong Kong, 83 Tat Chee Avenue, Kowloon 999077, Hong Kong, China; Department of Materials Physics, Saveetha School of Engineering, Saveetha Institute of Medical and Technical Sciences (SIMTS), Thandalam, Chennai 602105, Tamilnadu, India.

E-mail: [liuxinghui119@gmail.com](mailto:liuxinghui119@gmail.com)

**Funding information**

National Natural Science Foundation of China: 62264015, 52262042

National Science Foundation of Shaanxi Province: 2021JQ-635

Scientific Research Program of Yan'an University: YDQ2020-08

Scientific and Technological Innovation Team: 2017CXTD-01

**CHARACTERIZATION METHOD**

X-ray diffraction (XRD) was used to characterize the material's composition and internal atomic structure. Scanning electron microscopy (SEM) was used to study the prepared samples under an operating voltage (15 kV) to analyze the morphology and elemental composition of the prepared samples. The microstructure was characterized by transmission electron microscopy (TEM) with an accelerating voltage (200 kV). The Brunauer‒Emmett‒Teller (BET) method was applied to determine the specific surface area of the sample, and the sorption of N_2_ was used to study the evolution of the average pore diameter. The samples were measured by in situ irradiated X-ray photoelectron spectroscopy (ISIXPS) using an Al Kα source to characterize the element types, chemical valence states, chemical bonds, and other electronic structure information. The relevant energy spectra of the samples before and after illumination were detected by using axenon lamp (500 W) under normal temperature and pressure. The visible light response range of the sample was tested by ultraviolet‒visible diffuse reflectance spectroscopy (UV‒Vis DRS), and the band gap value of the sample can be obtained according to the formula (*αhν=A(hν-E_g_)^1/2^*). Photoluminescence (PL) was used to analyze the recombination rate of photogenerated electron-hole pairs. The time-resolved fluorescence data for samples were measured using the Edinburgh FLS1000. The excitation wavelength was set 325 nm. Transient photocurrent response curves were tested under a xenon lamp (300 W). Electrochemical impedance spectroscopy (EIS) was used to study the electron transport characteristics of the samples. Electron paramagnetic resonance (EPR) was used to verify the production of free radicals. In the EPR tests, free radicals were captured by configuring samples and 5,5-dimethyl-1-pyrroline N-oxide (DMPO) into suspensions in methanol and water, respectively. The settings for the EPR spectrometer were as follows: center field = 3505 G, sweep width = 100 G, microwave frequency = 9.843 GHz, and microwave power = 3.170 mW. The high-performance liquid chromatography‒mass spectrometry (HPLC‒MS) method was used to detect the intermediates of photocatalytic degradation to analyze the degradation pathway of pollutants. Inductively coupled plasma‒mass spectrometry (ICP‒MS) was used to measure the ionic strength of the solution after photocatalytic degradation. Total organic carbon was tested by varioTOC instrument of German ELEMENTAL. Total carbon content and total inorganic carbon content were tested, and the difference between the two was used to calculate the total inorganic carbon content.

**PREPARATION OF 2% La-Bi_2_WO_6_**

2% La-Bi_2_WO_6_ were prepared by the hydrothermal method. All the chemicals have not undergone any purification and were of analytical grade. The detailed preparation process was as follows: Bi(NO_3_)_3_⋅5H_2_O (2 mmol) was mixed with deionized water (80 mL) and ultrasonicated (15 min) to form solution A. After, a certain amount of La_2_O_3_ (0.04 mmol) was added to solution A and continuously stirred to form solution B. Following this, Na_2_WO_4_⋅2H_2_O (1 mmol) and Hexadecyl trimethyl ammonium Bromide (CTAB, 0.05 g) were dissolved in the solution B and agitated for 30 min at ambient temperature to form solution C. The solution C was moved into a Teflon-lined stainless vessel (100 mL) and kept at 120 ℃ for 24 h and then cooling naturally. Using deionized water and anhydrous ethanol to wash the resulting samples three times respectively. The products were dried at 80 ℃ for 10 h and marked 2% La-Bi_2_WO_6_.


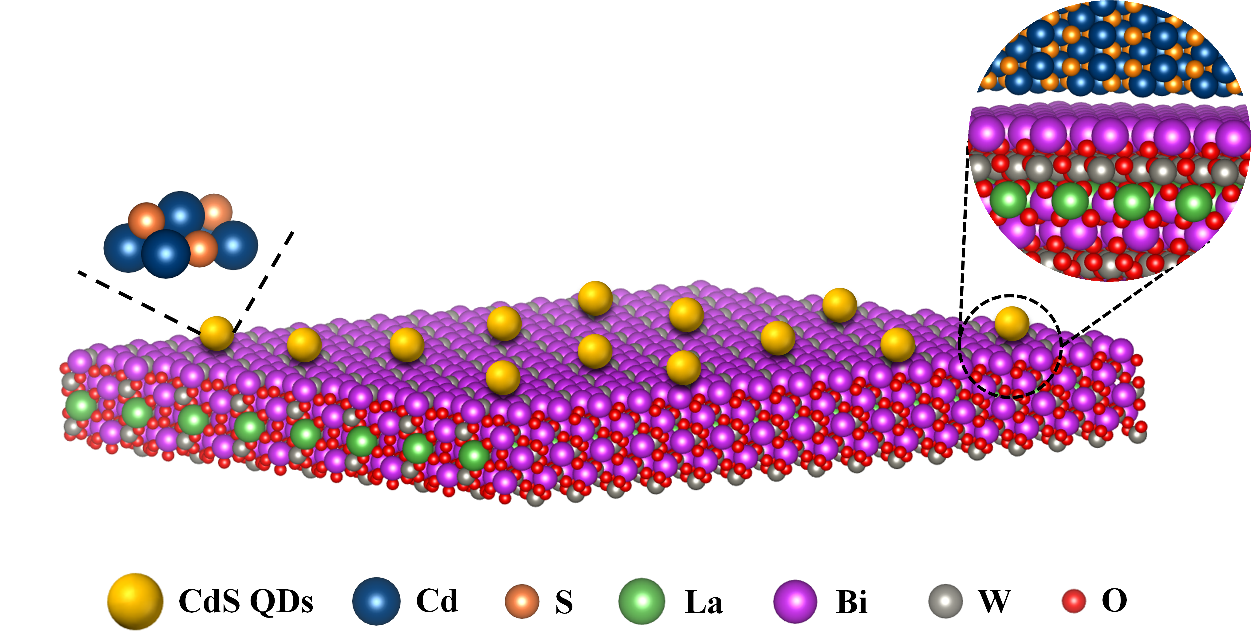


**FIGURE S1** The diagram of the heterojunction mode


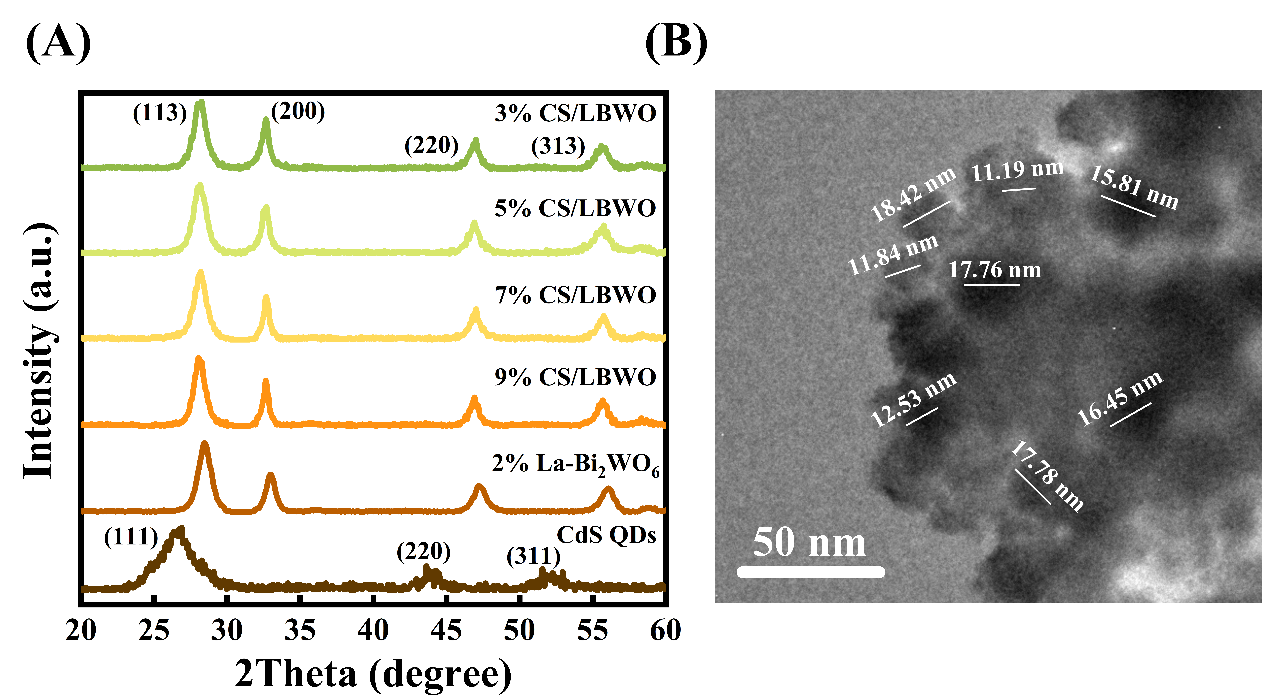


**FIGURE S2** (A)The X-ray diffraction of CS/LBWO (B)TEM diagram of CdS QDs


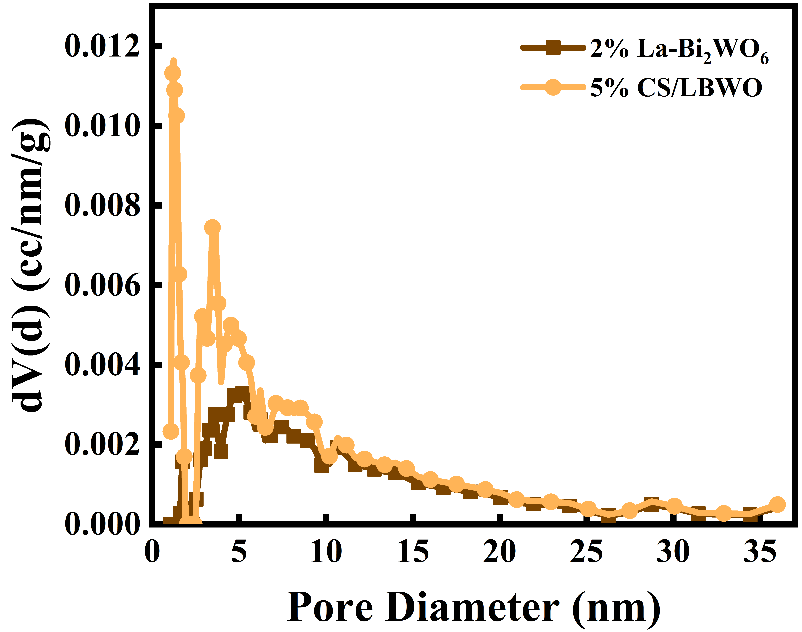


**FIGURE S3** pore size profiles of 2% La-Bi_2_WO_6_ and 5% CS/LBWO


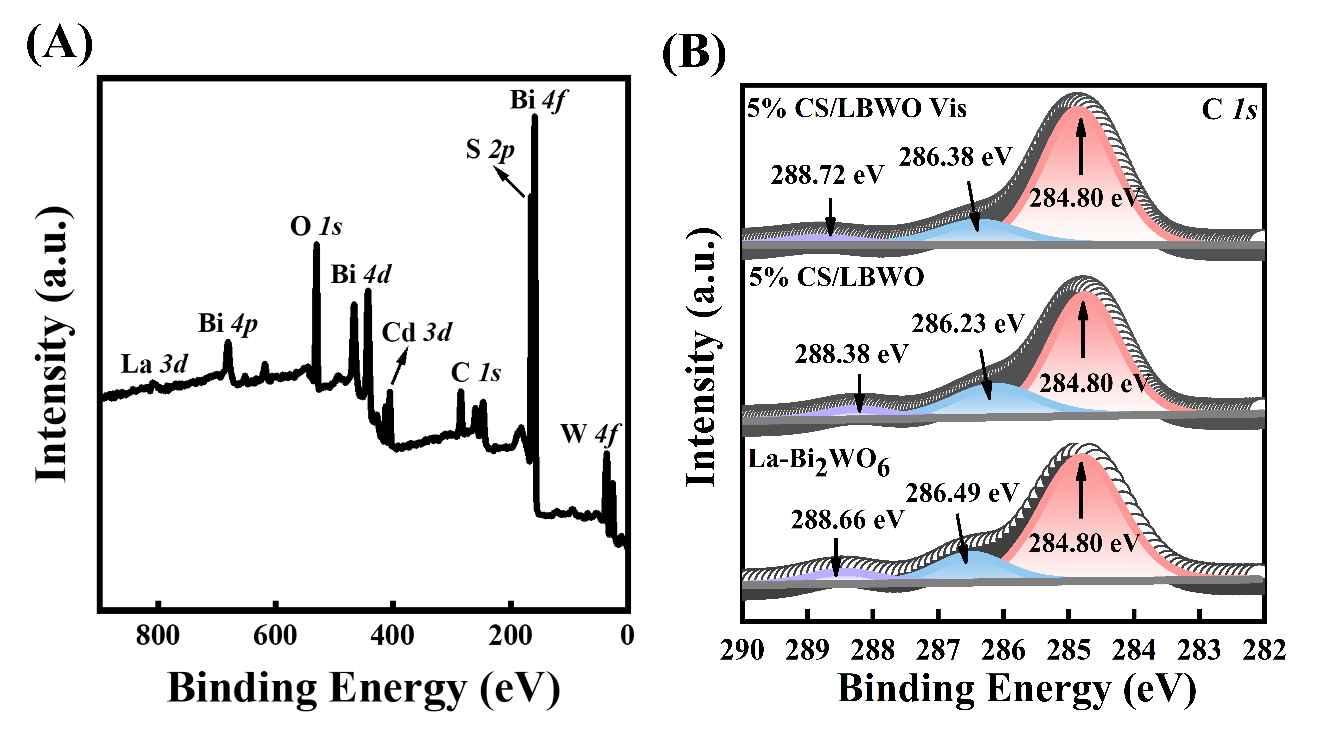


**FIGURE S4** The ISIXPS spectrum of CS/LBWO (A) Full spectra; (B) C *1s*.


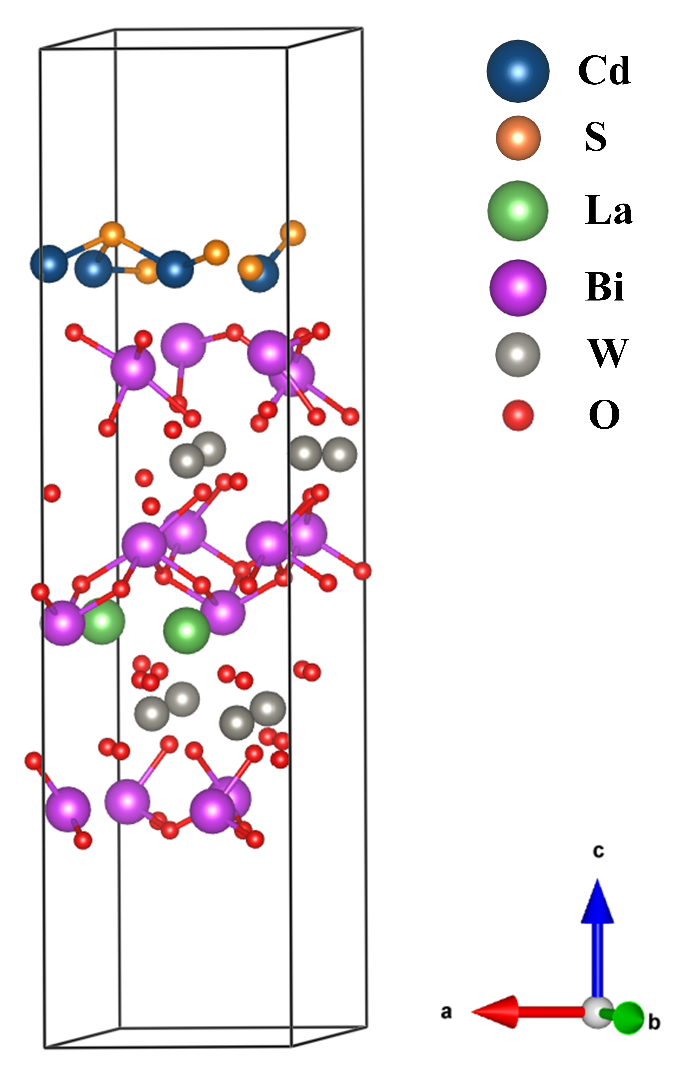


**FIGURE S5** The structure model of CS/LBWO.


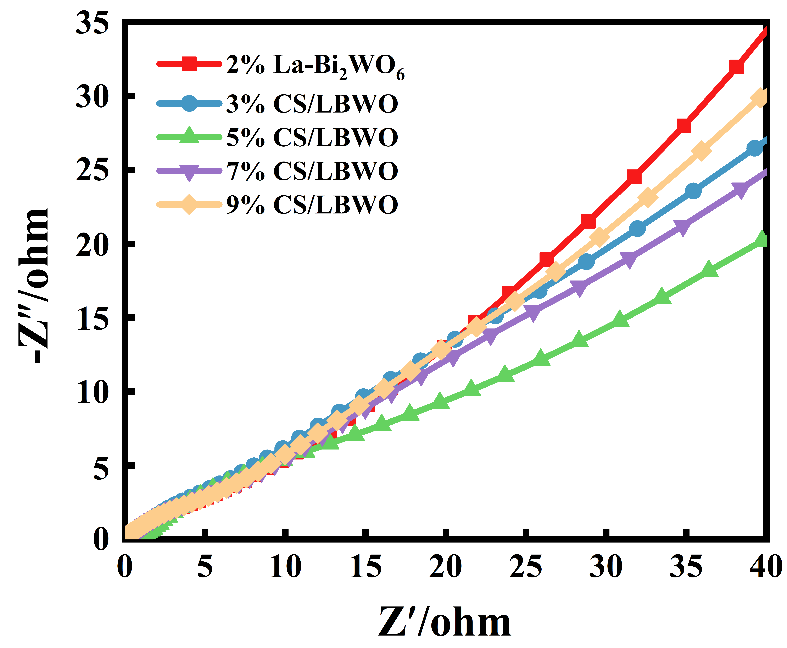


**FIGURE S6** The EIS conversion diagram for 2% La-Bi2WO6 and CS/LBWO.


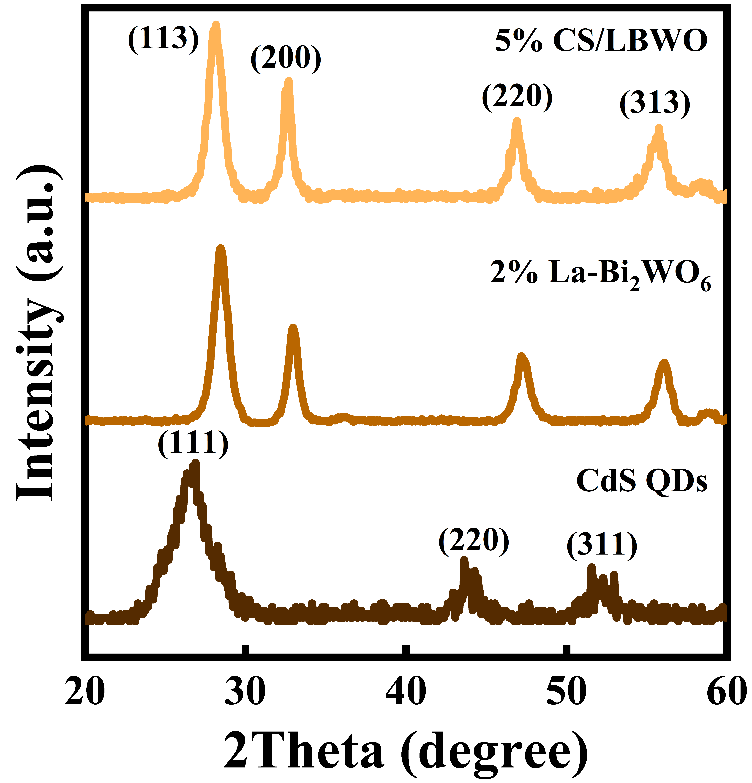


**FIGURE S7** The XRD before and after the degradation experiment.


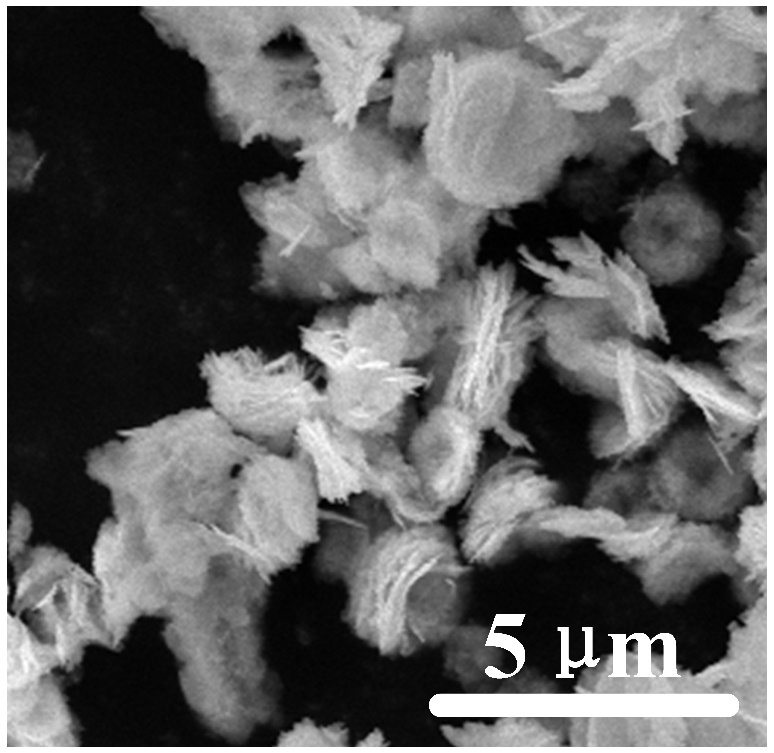


**FIGURE S8** The SEM before and after the degradation experimen

**TABLE S1** Related similar work of CS/LBWO

| Photocatalysts | Types | Pollutants | Degradation efficiency | DOI |
| --- | --- | --- | --- | --- |
| CdS/BiVO_4_ | Z | RhB  (10 mg L^-1^) | 60 min (94.70 %) | 10.1016/j.mssp.2020.105453 |
| F-CeO_2_/CdS | Ⅱ | RhB  (10 mg L^-1^) | 180 min (96.16 %) | 10.1016/j.colsurfa.2022.129256 |
| CdS/WO_3_ | Z | RhB  (5 mg L^-1^) | 120 min (90.50 %) | 10.1016/j.colsurfa.2018.10.033 |
| CdS/MoS_2_ | Ⅱ | RhB  (10 mg L^-1^) | 60 min (90.00 %) | 10.1016/j.ijhydene.2023.05.050 |
| BaTiO_3_/Bi_2_WO_6_ | Z | RhB  (5 mg L^-1^) | 90 min (99.90 %) | 10.1016/j.optmat.2021.110853 |
| BiOBr/Bi/Bi_2_WO_6_ | Z | RhB  (10 mg L^-1^) | 180 min (98.02 %) | 10.1016/j.optmat.2021.111641 |
| RGO/Bi_2_MoO_6_/Bi_2_WO_6_ | S | RhB  (10 mg L^-1^) | 120 min (98.53 %) | 10.1016/j.apsusc.2022.152788 |
| *CdS QDs/La-Bi_2_WO_6_* | ***S*** | ***RhB***  ***(10 mg L^-1^)*** | ***70 min (99.21 %)*** | ***This work*** |

**TABLE S2** TOC removal rate of 2% La-Bi_2_WO_6_ and 5% CS/LBWO

| Sample(Time) | TIC (mg L^-1^) | TC (mg L^-1^) | TOC (mg L^-1^) | Removal Rate |
| --- | --- | --- | --- | --- |
| Pure water(\) | 0.756 | 1.332 | 0.575 | \ |
| 2% La-Bi_2_WO_6_(0) | 0.637 | 18.731 | 18.094 | \ |
| 2% La-Bi_2_WO_6_(20) | 0.750 | 10.203 | 10.953 | 39.46 % |
| 2% La-Bi_2_WO_6_(40) | 1.504 | 6.487 | 7.991 | 55.83 % |
| 2% La-Bi_2_WO_6_(70) | 3.765 | 8.647 | 4.882 | 73.01 % |
| 5% CS/LBWO(0) | 0.608 | 18.560 | 17.952 | \ |
| 5% CS/LBWO(20) | 1.712 | 6.652 | 8.364 | 53.41 % |
| 5% CS/LBWO(40) | 2.697 | 7.315 | 4.618 | 74.27 % |
| 5% CS/LBWO(70) | 3.254 | 5.421 | 2.167 | 87.92 % |

TC: Total carbon; TIC: Total inorganic carbon; TOC: Total organic carbon.

(TOC=TC-TIC)

**TABLE S3** ICP-MS experiment of CS/LBWO

| Sample | [Intensity](javascript:;) | Concentration (mg/L) | Degradation Rate |
| --- | --- | --- | --- |
| 1 mg/L Cd^2+^ solution | 20414.82 | 1.0124 | \ |
| 3% CS/LBWO | 4328.21 | 0.2146 | 92 % |
| 5% CS/LBWO | 6417.18 | 0.3182 | 99 % |
| 7% CS/LBWO | 9395.36 | 0.4659 | 94 % |
| 9% CS/LBWO | 15513.02 | 0.7693 | 89 % |
| 5% CS/LBWO + Bentonite clay | 983.05 | 0.0487 | 94 % |

([Intensity](javascript:;)=20163.71*Concentration+1.081)
